# Tissue-Specific Microbiomes of the Red Sea Giant Clam *Tridacna maxima* Highlight Differential Abundance of Endozoicomonadaceae

**DOI:** 10.3389/fmicb.2019.02661

**Published:** 2019-11-26

**Authors:** Susann Rossbach, Anny Cardenas, Gabriela Perna, Carlos M. Duarte, Christian R. Voolstra

**Affiliations:** ^1^Red Sea Research Centre (RSRC) and Computational Bioscience Research Center (CBRC), Biological and Environmental Science and Engineering Division (BESE), King Abdullah University of Science and Technology (KAUST), Thuwal, Saudi Arabia; ^2^Department of Biology, University of Konstanz, Konstanz, Germany

**Keywords:** microbiome, metabarcoding, coral reef, symbiosis, giant clam, *Tridacna*

## Abstract

Giant clams (subfamily Tridacninae) are prevalent members of coral reef communities and engage in symbioses with algal photosymbionts of the family Symbiodiniaceae, similar to their scleractinian coral counterparts. However, we know little about their associated bacterial microbiome members. Here, we explored bacterial community diversity of digestive system, gill, and mantle tissues associated with the giant clam *Tridacna maxima* across a cross-shelf gradient (inshore, midshore, and offshore reef sites) in the central Red Sea using 16S rRNA gene amplicon sequencing. Different tissues harbor spatially stable and distinct microbial communities. Notably, diverse assemblages of bacteria affiliated to the family Endozoicomonadaceae were prevalent in all tissues, but particularly abundant in gills and to a lesser extent in digestive tissues. Besides Endozoicomonadaceae, bacteria in the families Pasteurellaceae, Alteromonadaceae, and Comamonadaceae were common associates, depending on the tissue queried. Taxonomy-based functional inference identified processes related to nitrogen cycling (among others) to be enriched in giant clam tissues and contributed by Endozoicomonadaceae. Our study highlights the tissue-specificity and broad taxonomic range of Endozoicomonadaceae associates, similar to other marine invertebrates, and suggests their contribution to nitrogen-related pathways. The investigation of bivalve-associated microbiome communities provides an important addition to the pathogen-focused studies for commercially important bivalves (e.g., oysters).

## Introduction

Tropical coral reefs are among the most productive ecosystems and provide important habitats for a diversity of organisms, including giant clams of the Tridacninae subfamily in the Indo-Pacific. Giant clams provide important ecosystem functions in coral reef ecosystems (Neo et al., 2015), including providing a food source for a number of predators and scavengers (Alcazar, 1986), shelter for commensal organisms (De Grave, 1999), or substrate for epibionts (Vicentuan-Cabaitan et al., 2014). Further, through their high photosynthetic (Jantzen et al., 2008; Rossbach et al., 2019) and calcification rates (Rossbach et al., 2019) they contribute to the primary production and spatial framework of the reef structure.

Similar to corals, Tridacninae engage in a symbiosis with unicellular algae of the family Symbiodiniaceae (Taylor, 1969; LaJeunesse et al., 2018) that are located in delicately branching tubules, which extend from the clam’s stomach into the outer mantle (Norton et al., 1992). Since comparable photosynthetic symbioses are very rare in other mollusks (Vermeij, 2013), Tridacninae stand out as a remarkable group of bivalves. While the symbiotic relationship of Tridacninae and Symbiodiniaceae is a prominent field of study (e.g. Taylor, 1969; Fitt et al., 1986, 1995; Weber, 2009; DeBoer et al., 2012; Pappas et al., 2017), comparatively little is known about the bacteria associated with giant clams, albeit the importance of bacteria to animal and plant function is now widely acknowledged (McFall-Ngai et al., 2013; Bang et al., 2018). For other reef organisms, such as corals and sponges, patterns of microbial diversity and even presumptive functions are broadly explored (Ainsworth et al., 2010; Bourne et al., 2016; Neave et al., 2017a,b). It should be pointed out that, although research on bacterial communities associated with bivalve species dates back more than 100 years (Round, 1914, 1916; Bates and Round, 1916), studies mainly focused on commercially important species, such as *Crassostrea* spp., *Mytilus* spp., and *Venerupis* spp. (Meisterhans et al., 2016; Pierce et al., 2016; Pierce and Ward, 2018; Vezzulli et al., 2018). Further, because of the economic value and consumption of these species by humans, the majority of the literature focuses on the impact of bacterial pathogens (Romalde et al., 2014; Wendling et al., 2014), in particular *Vibrio* spp. (Pruzzo et al., 2005; Beaz-Hidalgo et al., 2010; Romalde et al., 2014), rather than targeting microbial diversity patterns.

Here, we addressed this gap in our understanding of Tridacninae microbiomes by characterizing the bacterial microbiome associated with different tissues of Red Sea *Tridacna maxima*. Tridacninae are one of the most prevalent bivalve groups in Red Sea coral reefs (Zuschin et al., 2000, 2001). The most common giant clam (Roa-Quiaoit, 2005; Ullmann, 2013) is *T. maxima*. This species has also been reported to have the broadest geographical distribution (Ayala et al., 1973), reaching from East Africa to the Central Pacific, and therefore comprises almost the entire geographical range of all other giant clam species (bin Othman et al., 2010).

We assessed *T. maxima* across reef sites in the central Red Sea following a cross-shelf gradient and used 16S rRNA gene amplicon sequencing to characterize the bacterial community composition of three tissues (digestive system, gills, and mantle).

## Materials and Methods

### Sample Collection

In May 2018, 18 *T. maxima* specimens (shell length of 15 ± 1 cm; mean ± SD), were collected in a water depth of about 3 m at three different reef sites nearby King Abdullah University of Science and Technology (KAUST). Six clams were sampled at each of one inshore reef (Fsar, 22° 14′27” N, 39° 02′ 51″ E), one midshore reef (Al Fahal, 22° 15′ 2” N, 38° 57 ‘45″ E), and one offshore reef (Shib Nizar, 22° 19′ 20” N, 38° 51′ 26″ E), respectively, for a total of 18 clams. Clams were stored individually in seawater-filled plastic bags, to avoid cross-contamination and placed in a cooler filled with seawater. In addition, seawater samples were collected at the respective reefs at the same depth using 9 L polycarbonate carboy containers (Nalgene, Thermo Scientific Fisher, USA). The containers were kept in a cooling box on ice until arrival at the laboratory, where, for each reef respectively, 2 L of seawater were immediately filtered through a 0.22 μm Hydrophilic Polyvinylidene Fluoride (GVWP) filter (Millipore, Merck KGaA, Darmstadt, Germany) using a peristaltic pump. Filters were subsequently frozen at −20°C.

### Clam Dissection, Tissue Homogenization, DNA Isolation and Sequencing

The *Tridacna* specimens were cut open with a scalpel, and tissue samples of three different compartment were collected: gills, mantle, and the digestive system. All samples were weighed to the nearest 0.001 mg. The clam tissue was then homogenized using an Ultra Turrax T18 homogenizer (IKA-Werke GmbH and Co. KG, Staufen, Germany) with 15 ml MilliQ water (sterilized under UV light for 1 h, and filtered through a 0.2 μm syringe filter). The homogenate was frozen at −80°C until further processing.

In total, we extracted DNA from 62 samples, corresponding to 54 *T. maxima* tissue samples, 3 seawater samples, and 4 negative controls (3 controls to assess for kit and lab contaminations and 1 negative control, which originated from the subsequent PCRs). DNA from water and tissue samples were extracted with the Qiagen DNeasy Blood & Tissue Kit (Qiagen, Hilden, Germany) following the manufacturer’s instructions with minor modifications. Briefly, 90 μl of the tissue homogenate were added to 1.5 ml Eppendorf tubes containing 90 μl of ATL buffer and 20 μl of Proteinase K and were incubated at 56°C for 1 h. DNA extractions were then conducted according to manufacturer’s instructions. Water filters were thawed and placed in 1.5 ml Eppendorf tubes, 360 μl of ATL buffer and 40 μl Proteinase K buffer were added, and the tubes were incubated at 56°C for 20 min. DNA extractions were then conducted according to manufacturer’s instructions. DNA concentrations were measured using a Qubit 2.0 fluorometer (Thermo Fisher Scientific), and samples were adjusted to 10 ng/μl. To amplify the hypervariable regions V5 and V6 of the bacterial 16S rRNA gene, the primers 784F [5′-TCGTCGGCAGCGTCAGATGTGTATAAGAGACAGAGGATTAGATACCCTGGTA-3′] and 1061R [5′-GTCTCGTGGGCTCGGAGATGTGTATAAGAGACAGCRRCACGAGCTGACGAC-3′] (Illumina adapter sequences underlined) were used (Andersson et al., 2008). For all samples, triplicate PCRs were performed using 10 ng of DNA using the Qiagen Multiplex PCR kit and a final primer concentration of 0.3 μM in a reaction volume of 10 μl. Thermal cycling conditions were: 95°C for 15 min, followed by 27 cycles of 95°C for 30 s, 55°C for 90 s, 72°C for 30 s, and a final extension cycle of 72°C for 10 min. About 5 μl of the PCR were run on a 1% agarose gel to visualize amplification. Triplicated PCRs from each sample were pooled, cleaned with ExoProStar 1-step (GE Healthcare, UK), and subsequently underwent an indexing PCR step (8 cycles, according to the Illumina 16S metagenomic sequencing library preparation protocol). Successful addition of indexes was checked by size comparison on a 1% agarose gel. Samples were purified and normalized using the SequalPrep Normalization Plate Kit (Invitrogen, Carlsbad, USA). Next, from each pooled and normalized sample, 4 μl were transferred into a 1.5 ml Eppendorf tube, and concentrated using the CentriVap Benchtop Vacuum Concentrator (Labconco, USA). The final library was assessed using a Bioanalyzer (Agilent Technologies, USA) and quantified using the Qubit dsDNA HS assay with the Qubit® 2.0 Fluorometer (Thermo Fisher Scientific, USA). The 16S rRNA gene amplicon library was sequenced at the KAUST sequencing facility on the Illumina MiSeq platform. Sequencing was performed at 6 pM with 20% PhiX on the Illumina MiSeq platform at 2 × 301 bp paired-end V3 chemistry according to the manufacturer’s specifications.

### Bacterial Community Analysis

Sequence reads were processed using mothur v.1.39.5 (Schloss et al., 2009). In brief, after quality filtering and adaptor trimming, paired-end reads were merged to contigs, and sequences were aligned against the SILVA database (version 132) using “align.seqs”. Chimeric sequences were removed using USEARCH (Edgar, 2017) and taxonomically classified using the Greengenes database (release gg_13_5_99, May 2013) (DeSantis et al., 2006). The Greengenes database was used, since it contains substantially more bacterial sequences from coral studies than other databases and PICRUSt relies on the Greengenes taxonomy (see below). OTUs assigned as contaminants (i.e., were present in the negative controls) were removed. An OTU was considered a contaminant when the ratio of the sum of its relative abundance in the negative control samples over the sum of relative abundances in all other samples was greater than 0.1). OTU sequences that were classified as “Endozoicimonaceae” in Greengenes (39 OTUs) were confirmed to belong to the recently proposed family Endozoicomonadaceae (Bartz et al., 2018) using SILVA. Singletons as well as mitochondrial and chloroplast sequences were removed, prior to applying a 97% similarity cutoff for OTU clustering.

Chao1 richness (Chao and Chiu, 2001) and inverse Simpson indices (Simpson, 1949) were calculated using the R package Vegan (Oksanen et al., 2013). Evenness was calculated using the R package fundiv (Gagic et al., 2015). To calculate weighted UniFrac (Lozupone and Knight, 2005) and Faith’s phylogenetical distances (Faith, 1992), OTU sequences were aligned using MAFFT and a maximum-likelihood tree was build using RAxML software v.8.0.26 (Stamatakis, 2006) with the GTRCAT model and 1,000 bootstraps. Faith’s phylogenetical distances were calculated using the function phyloseq_phylo_div from the R package PhyloMeasures (Tsirogiannis and Sandel, 2016) and weighted UniFracs were calculated using the “UniFrac” function from the phyloseq package.

Rarefaction curves were plotted using the ggrare function. A permutational multivariate analysis of variance was done using “adonis” function on dissimilarity Bray-Curtis distances of the OTU set to test for differences between tissues. Pairwise adonis test was conducted using an R wrapper function for multilevel pairwise comparison (Martinez Arbizu, 2019). Normal distribution was tested using Shapiro-Wilk test, and homogeneity of variances were evaluated using the function “betadisper” from Vegan package. Kruskal-Wallis rank and pairwise Wilcoxon sum tests were used to evaluate significant differences in alpha-diversity indexes. All bioinformatic workflows can be assessed at https://github.com/ajcardenasb/Tridacna_microbiome.

### Phylogenetic Analysis of OTUs Affiliated With the Family Endozoicomonadaceae

A total of 39 OTUs classified as Endozoicomonadaceae, in addition to selected bacterial reference strains (Supplementary Table S1), were aligned using MAFFT and a maximum-likelihood tree was built as described above and visualized in the R package “phyloseq” (McMurdie and Holmes, 2013) to annotate abundance and prevalence across samples.

### Taxonomy-Based Functional Prediction

OTU taxonomic affiliations were used to determine the nearest genome-sequenced taxon, which was then used to infer the abundance of KEGG orthologs from normalized OTU abundances using PiCRUSt (Langille et al., 2013). We used PICRUSt’s Nearest Sequenced Taxon Index (NSTI) to quantify the relatedness of each taxa within a sample to the closest related available genome representative. NSTI is based on substitutions per site in the 16S rRNA gene and weighted by the abundance of that organism in the community. NSTI scores are therefore a proxy to assess the accuracy of PICRUSt functional prediction. KEGG ortholog abundance was aggregated into KEGG modules using a mapping file available from the KEGG API. KEGG module abundance matrix was used as input to LEfSE to identify functional features that are different between tissues (Segata et al., 2011). In brief, LEfSE implements a non-parametric factorial Kruskal-Wallis test to determine significant differential abundance of KEGG modules between tissues and a linear discrimination analysis (LDA) to estimate the effect size of each differentially abundant KEGG module. Pairwise comparison of KEGG modules with *p*-values <0.05 and effect sizes >2 were considered significant. In addition, we focused only on KEGG modules contained in the reference pathway “Microbial metabolism in diverse environments” (map01120).

## Results

### Bacterial Community Composition of *Tridacna maxima*

**Figure 1:**
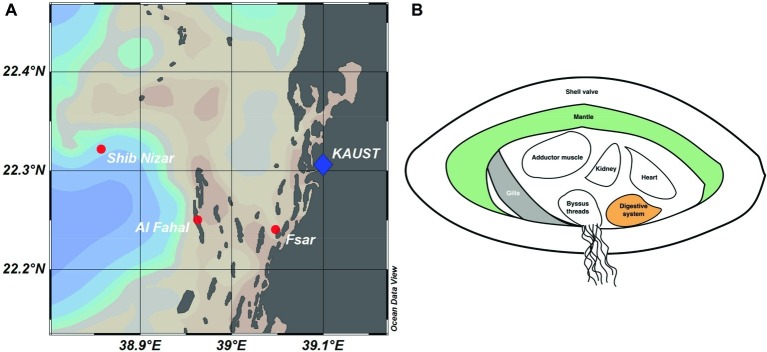
Reef locations and *Tridacna maxima* tissue sampling in the Central Red Sea. **(A)** Map showing sampled reef sites (inshore – Fsar reef, midshore – Al Fahal reef, offshore – Shib Nizar reef). **(B)** Schematic cross section of *T. maxima* indicating the tissue compartments sampled: Digestive system (orange), gills (gray), and mantle (green).

We sequenced 57 16S rRNA gene amplicon libraries comprising a total of 2,480,709 sequences, with a mean (±SD) of 40,011 ± 26,036 sequences per sample and an average length of 309 bp, for subsequent analyses (Supplementary Table S2). The dataset comprised three tissue compartments (digestive system, gills, and mantle) of 18 *T. maxima* specimens from three reef sites across a shelf-gradient (inshore, midshore, offshore) (Figure 1). In addition, seawater was collected from each reef site. Thus, a total of 6 specimens × 3 sites × 3 tissue compartments = 54 + 3 seawater samples were analyzed.

**Figure 2:**
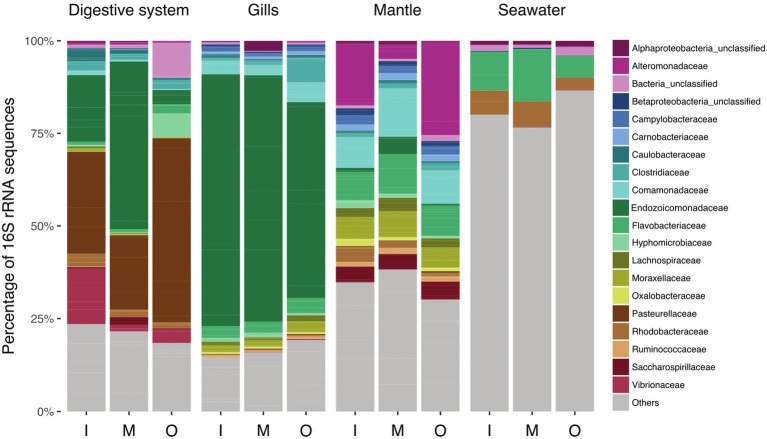
Taxonomy bar plot of bacterial communities associated with *T. maxima* tissues. Bar plots show averaged relative abundances of the 20 most abundant families for the three tissue compartments (digestive system, gills, and mantle) over reef sites (I, inshore; M, midshore; O, offshore) and seawater samples from the three reef sites.

**Figure 3:**
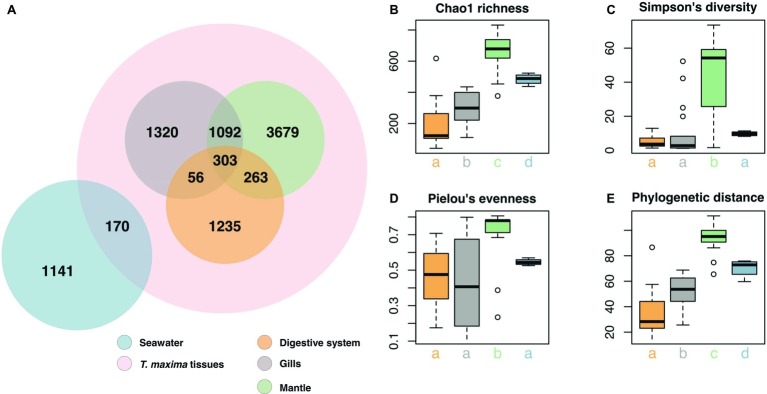
Bacterial community composition of *T. maxima* tissues and seawater. **(A)** Distribution of the 9,259 OTUs over tissue types and seawater. Compartments sampled are color-coded: Seawater (blue), overall clam samples (red) with digestive system (orange), gills (gray) and mantle (green). **(B–E)** Alpha diversity estimates of clam tissues and seawater with **(B)** Chao1 richness **(C)** Simpson’s diversity index. **(D)** Pielou’s evenness. **(E)** Faith’s phylogenetic distance. Letters denote statistical significance (*p* < 0.05) evaluated using Kruskal-Wallis test and Wilcoxon pairwise comparisons.

Clustering of sequences into operational taxonomic units (OTUs) yielded 9,259 bacterial taxa (Figure 2, Supplementary Data). Of these, 7,948 OTUs were present exclusively in the clam, in at least one of the tissues, 1,141 OTUs were present in seawater only, and a small (2%) fraction (170 OTUs) were present in both, seawater and giant clam tissues (Figure 3A). Comparison of microbial communities across reefs showed that microbiome structure of *T. maxima* did not statistically differ between sites (PERMANOVA, *p* = 0.121, *F* = 1.34, *R*^2^ = 0.03; Supplementary Table S3). Further, we found no significant interaction between reef site and tissue type (PERMANOVA, *p* = 0.638, *F* = 0.93, *R*^2^ = 0.06; Supplementary Table S3). Conversely, seawater bacterial communities differed significantly from *T. maxima* bacterial communities, and importantly, the three respective tissue types differed significantly from each other (PERMANOVA, *p* = 0.001, *F* = 12.24, *R*^2^ = 0.40; Supplementary Tables S3, S4). Thus, bacterial community structure was conserved across reef sites (Figure 2), and for subsequent analyses same tissue types were pooled over reef sites.

Taxonomic community composition of the top 20 families by reef site (Figure 2; for the sample-based plot see Supplementary Figure S1) revealed a number of bacterial families to be highly represented in clam tissues, including Pasteurellaceae, Alteromonadaceae, Comamonadaceae, and Endozoicomonadaceae. Notably, bacterial communities of the mantle tissue were more diverse at the family level considering the top 20 families. In comparison, digestive system and gills were dominated by fewer bacterial families, such as Pasteurellaceae and Endozoicomonadaceae. Notably, we found a strikingly high abundance of Endozoicomonadaceae in the gill tissues.

Overall, the three tissue compartments harbored different bacterial OTUs and shared only 303 (3.8% of *Tridacna* OTUs) (Figure 3A). Although bacterial communities of gill and mantle tissues showed some degree of similarity (with 1,092 OTUs shared between the two of them) (Figure 3A), we identified 1,320 OTUs that were only present in the gills. While the digestive system shared the lowest number of OTUs with the gill and mantle tissues (56 and 263 OTUs, respectively), mantle tissues displayed the highest richness with 5,337 OTUs (Figures 3A,B). The bacterial community from the mantle also showed the most diverse community (Figure 3C), the highest levels of evenness (Figure 3D) (Pairwise Wilcoxon test, *p* < 0.05; Supplementary Table S5), and the highest phylogenetic relatedness between OTUs (Figure 3E) (Pairwise Wilcoxon test, *p* < 0.005; Supplementary Table S5). Bacterial communities associated with the digestive system were the least diverse (1,857 OTUs) and showed significantly lower richness (Pairwise Wilcoxon test, *p* < 0.05; Supplementary Table S5) (Figures 3B,C).

### Diversity of Endozoicomonadaceae

**Figure 4:**
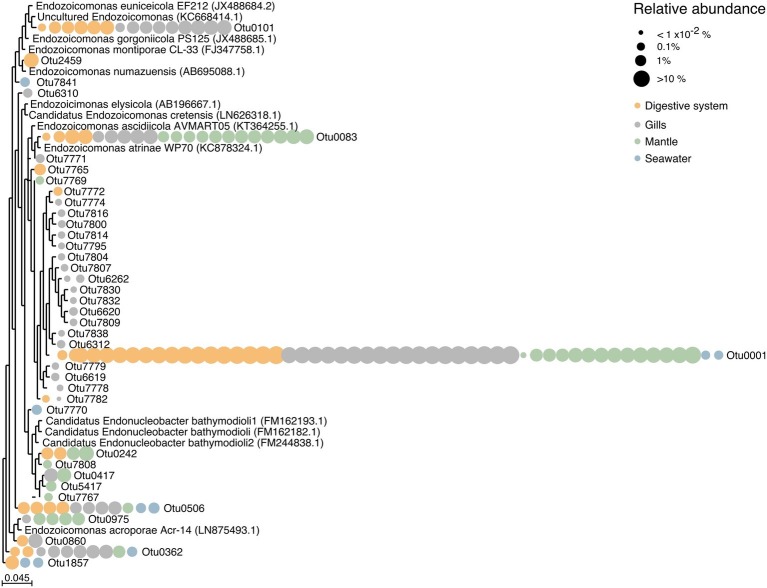
Phylogenetic relationship among Endozoicomonadaceae OTUs from the giant clam *T. maxima.* Maximum-likelihood phylogenetic tree of *T. maxima*-associated Endozoicomonadaceae OTUs and reference Endozoicomonadaceae strains from other marine invertebrates. Color-coded dots denotate the tissue in which each OTU was present. Relative abundances of OTUs are visualized by dot size, reaching from >10% (big dots) to <1 × 10^−2^% (smallest dots). The scale bar corresponds to the estimated number of nucleotide changes per branch length.

We found a large number of OTUs assigned to the family Endozoicomonadaceae (*n* = 39), but only few of those were highly abundant (Figure 4, Supplementary Table S6). Notably, the majority of Endozoicomonadaceae were tissue-specific (25 OTUs), with only a few (4 OTUs) present in all three tissues, including the most abundant bacterial taxon OTU0001. Interestingly, only three Endozoicomonadaceae OTUs were found in seawater.

Overall, tissue-specific Endozoicomonadaceae OTUs showed on average lower abundances than those present in all three tissue compartments (Figure 4, Supplementary Table S6). The gills harbored the greatest variety of Endozoicomonadaceae OTUs, 19 of which were exclusively found in this tissue, while 4 OTUs were exclusively found in the digestive system, and 4 exclusively in the mantle. When comparing the taxonomic relatedness of clam-associated Endozoicomonadaceae OTUs with those previously sequenced/cultured, we found that associated Endozoicomonadaceae were related to a broad range, previously reported from other environment and host animals. Among those were *Endozoicomonas ascidiicola* [previously described in the pharynx of Mediterranean and Scandinavian ascidians (Schreiber et al., 2016)], *Endozoicomonas euniceicola* [reported from octocorals *Eunicea fusca* and *Plexaura* spp. (Pike et al., 2013)], and *Endozoicomonas acroporae* [sequenced from whole body homogenate samples of *Acropora* spp. (Sheu et al., 2017)] (Supplementary Table S1). We observed greatest phylogenetic distances between the highest abundant OTUs (i.e., between OTU0001, OTU0101, OTU0083, OTU0506, OTU0562). The majority of tissue-specific Endozoicomonadaceae were associated with the gills.

### Predictive Functional Profiling of Associated Bacteria

**Figure 5:**
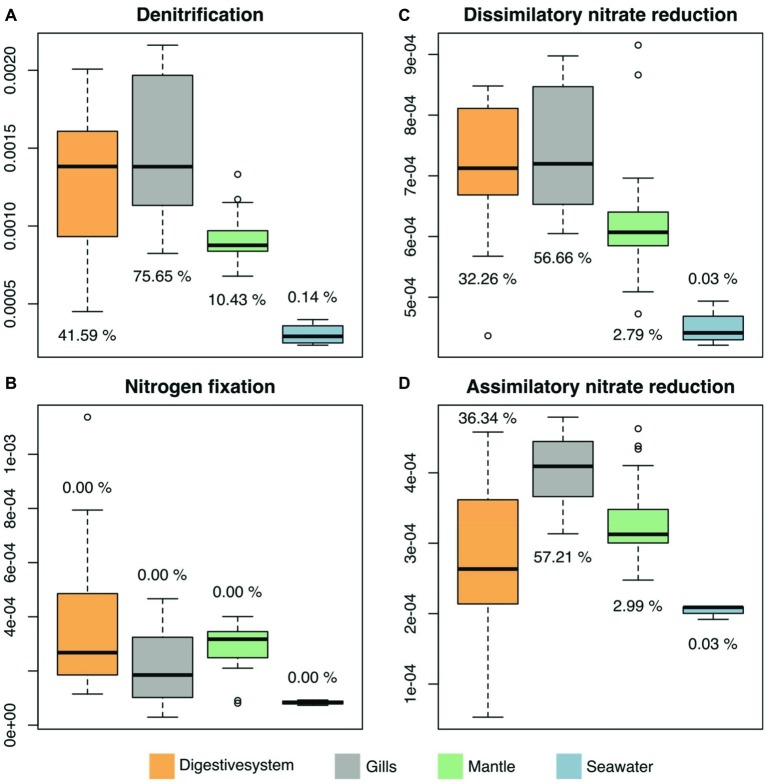
Predicted microbiome functional features of nitrogen cycling related processes across tissues of the giant clam *T. maxima* and seawater. Shown are **(A)** denitrification, **(B)** nitrogen fixation, as well as **(C)** dissimilatory and **(D)** assimilatory nitrate reduction. Tissue compartments are color-coded (orange- digestive system, gray – gills, green – mantle tissues of *T. maxima*, blue - seawater). Percentages shown denote the relative contribution of Endozoicomonadaceae OTUs.

We predicted functional profiles from the bacterial diversity data using PICRUSt to test whether differences in bacterial diversity between tissues may translate into putative functional differences. Average NSTI scores were found to be in an acceptable range for metagenomic predictions (mean NSTI digestive system = 0.102, gills = 0.11, mantle = 0.08; Supplementary Figure S2) (Langille et al., 2013; Mizrahi-Man et al., 2013). We considered only processes that displayed significant differences between the three investigated tissues and the surrounding seawater. To do this, we used LEfSE, considering a set of 249 KEGG modules in the reference pathway “*Microbial metabolism in diverse environments*” (map01120), with an effect size higher than 2, which produced 25 modules for further consideration (Supplementary Figure S3, Supplementary Table S7). Among these 25 modules, 4 processes were related to nitrogen (N) cycling pathways (Figure 5). Arguably, those are particularly important in oligotrophic oceans, which is why we assessed the contribution of underlying taxa to N cycling pathways (i.e., denitrification, nitrogen fixation, dissimilatory nitrate reduction, and assimilatory nitrate reduction), which identified bacteria in the family Endozoicomonadaceae as a main contributor, in particular in the gills and the digestive system (Figure 5, Supplementary Table S8).

## Discussion

Bacterial communities of *T. maxima* were highly consistent and associated bacterial families were highly similar between replicates for tissue compartments across sampling locations in our study. This is in contrast to studies on other bivalves, such as oysters, that reported high variations between specimens (e.g. King et al., 2012; Lokmer et al., 2016; Pierce and Ward, 2018), and scleractinian corals from the same region (Central Red Sea), which show pronounced differences in bacterial community composition across locations (Roder et al., 2015). In contrast, the bacterial microbiome of the giant clam *T. maxima* is highly consistent across the cross-shelf gradient sampled.

*T. maxima* tissue has a much higher bacterial richness, by almost one order of magnitude, than the surrounding seawater samples (8,118 OTUs in *T. maxima* vs. 1,311 OTUs in seawater, respectively). Substantial differences between the bacterial community of the surrounding seawater and bivalve hosts (e.g., *Crassostrea gigas* and *Mytilus galloprovincialis*) were previously reported (Lokmer et al., 2016; Vezzulli et al., 2018). However, observations vary, from cases of higher diversities in the bivalve associated bacterial communities (Vezzulli et al., 2018) to higher species richness in seawater samples (Lokmer et al., 2016). Moreover, *T. maxima* displays a remarkably rich bacterial community, exceeding richness reported on coral species from the Central Red Sea by 4- to 30- fold when comparing 8,118 OTUs reported here in *T. maxima* to ~300 OTUs in *Stylophora pistillata* (Bayer et al., 2013) to ~2,000 OTUs in *Pocillopora verrucosa* (Pogoreutz et al., 2018). One component of the high bacterial species richness in *T. maxima* may come from the strong specialization and differentiation among the three sampled tissue compartments, suggesting that microbes in these tissues are highly selected and functionally distinct. So far, bacteria associated with bivalves were reported to be utilized as a food source (Harris, 1993), to assist the digestion of food (Prieur et al., 1990), or even supply the host with vitamins and amino acids (Newton et al., 2008). They may also protect their bivalve host from other, potentially harmful bacterial groups by either preventing the settlement of these putative pathogens by growing in high densities (Pujalte et al., 1999) or by producing antimicrobial agents (Castro et al., 2002).

Our results show that the digestive system has the lowest microbial richness among all three compartments. This is somewhat surprising as the digestive system, given its respective function, may be expected to comprise bacteria from ingested food. In general, microbes, located in the digestive system, encounter a comparatively extreme environment (i.e., low pH) (Pierce and Ward, 2018), which may result in specialization of bacteria associated to this compartment, although phylogenetic diversity does not necessarily correlate with functional diversity (Burke et al., 2011). Among all three tissue compartments, the mantle of *T. maxima* displayed not only the most diverse bacterial community, showing a high evenness, but also the highest species richness and phylogenetic distance. This potentially high specificity of bacteria associated with the mantle is reflected by the fact that this tissue only shares very few OTUs with seawater, although mantle tissues are directly exposed to the surrounding water column. These results confirm that a separate assessment of bacterial communities associated with different compartments in bivalves supports a better overall understanding of bacterial distributions in animal hosts (Pollock et al., 2018).

Tissue-associated *T. maxima* bacterial communities harbor a number of bacterial families, including Pasteurellaceae, Alteromonadaceae, Comamonadaceae, and Endozoicomonadaceae. Among these families especially the high abundance of bacteria in the family Endozoicomonadaceae, including the most abundant OTU (OTU0001), is striking. This family was found in all three giant clam compartments, with particularly high abundances in the gill tissues. Endozoicomonadaceae have previously been reported from a number of marine organisms (Neave et al., 2017a), from simple invertebrates, like sponges (Bartz et al., 2018) and corals (Bayer et al., 2013; Neave et al., 2017a; Pogoreutz et al., 2018) to complex vertebrates like fish (Katharios et al., 2015). For marine invertebrates, including giant clams, it has been reported that the presence of Endozoicomonadaceae-related sequences (referred to as Oceanospirillales) correlates with the association with photosynthetic symbionts such as Symbiodiniaceae (Bourne et al., 2013). In line with these findings, we observed a high number of distinct Endozoicomonadaceae taxa in *T. maxima.* However, unlike to the previously reported phylogenetic pattern, in which the association with Endozoicomonadaceae correlates with the presence of symbionts, highest abundances of this family were found within the gill tissues of *T. maxima*, which are the only compartment among the three investigated not harboring Symbiodiniaceae. In fact, there is an apparent overall dominance of Endozoicomonadaceae in *T. maxima*, but we observed also distinct differences across tissues. High abundances of Endozoicomonadaceae have been previously also reported for tissues of other bivalves, such as intestines of the comb pen shell *Atrina pectinate* (Hyun et al., 2014), the gills of the deep-water bivalve *Acesta excavate* (Jensen et al., 2010), as well as both, gills and intestinal content of the blue mussel *Mytilus edulis* (Schill et al., 2017). For the latter, the authors also reported tissue-dependent abundances of Endozoicomonadaceae (sequences assessed from gills and intestinal content comprised up to 67.6 and 37.2%, respectively of one OTU representing this family).

Notably, only 8 out of the total 39 observed Endozoicomonadaceae OTUs were present in more than one tissue (including the most abundant OTU0001), and the majority of Endozoicomonadaceae OTUs were tissue-specific. Among all three tissues, the gills of *T. maxima* harbored the greatest number of Endozoicomonadaceae OTUs (19), which were exclusively found in this tissue, while only 4 Endozoicomonadaceae OTUs were each associated with the digestive system and mantle tissues. The four most abundant Endozoicomonadaceae OTUs harbored by the digestive system were also found in the gill tissues, which may suggest that they ended up in the digestive system due to accidental digestion of bacteria from the gills. However, we deem this to be unlikely, given that a total of eight Endozoicomonas-related OTUs found in this study were observed in the digestive system but not in the gills, with one of them being OTU242, the fifth abundant Endozoicomonas OTU overall.

Some of the observed Endozoicomonadaceae OTUs were found to be related to previously described OTUs. Among those were OTU083 (found in digestive, gill, and mantle tissues of *T. maxima*) and OTU0101 (found in digestive and gill tissues of *T. maxima*) to *Endozoicomonas ascidiicola* (Schreiber et al., 2016) and *Endozoicomonas euniceicola* (Pike et al., 2013), respectively, which have been both previously reported for the octocorals *Eunicea fusca* and *Plexaura* spp., as well as OTU0975 (present in mantle tissues of *T. maxima*) and *Endozoicomonas acroporae* (Sheu et al., 2017).

Although Endozoicomonadaceae bacteria are globally distributed and have been shown to be often abundantly associated with their hosts, their functional role(s) remain(s) unknown. Yet, there have been a number of functions proposed for this genus, both beneficial and harmful, including nutrient acquisition and cycling (Bourne et al., 2013; Correa et al., 2013; Neave et al., 2017a), a contribution to host health (Bayer et al., 2013; Jessen et al., 2013; Roder et al., 2015) as well as structuring of the host microbiome (Bourne et al., 2013).

We found overall high abundances of Endozoicomonadaceae bacteria across the three *T. maxima* tissues investigated. In particular, the gill tissue showed high diversification in associated Endozoicomonadaceae, which are phylogenetically related to OTU0001. The gills of giant clams have been previously shown to be responsible for the uptake and assimilation of ammonia from the surrounding seawater (Rees et al., 1994; Shepherd et al., 1999). The ability of assimilating exogenous ammonia and recycling nitrogen between the giant clam host and their symbionts is assumed to be reason for the success of Tridacninae in the oligotrophic waters of tropical oceans (Boo et al., 2017). The strong association of Endozoicomonadaceae with giant clam gill tissues could therefore be an indication for the contribution of this bacterial group to processes related to nitrogen uptake. This hypothesis was further supported by our functional attribution analysis, which showed that OTUs associated with processes related to nitrogen were prevalent in all tissues, but showed particularly high levels in gill tissues. Relatively high abundances of Endozoicomonadaceae in the gill tissues have been previously also reported for two Red Sea oyster species, *Chama savignyi* (Zurel et al., 2011) and *Spondylus spinosus* (Roterman et al., 2015).

Mapping of genes associated with nitrogen-related processes to their bacterial taxonomic affiliation revealed the dominance of Endozoicomonadaceae. This further correlates with the high abundance and distinct Endozoicomonadaceae OTUs found in the gills of giant clams in our study. Although predictive functional profiling should be considered with caution, we hypothesize that this bacterial family may play a role in nitrogen-related processes of giant clam holobionts. It should be pointed out that Endozoicomonadaceae contributed to dissimilatory nitrate reduction, which refers to the anaerobic respiration using nitrate as electron acceptor producing ammonium. As such, Endozoicomonadaceae may assist their animal hosts in making nitrogen sources in nutrient poor environments available that are otherwise inaccessible. This hypothesis is further supported by the fact that a previous study (Neave et al., 2017a), examining the genomes of other Endozoicomonadaceae isolated from marine invertebrates (*E. elysicola* (Kurahashi and Yokota, 2007), *E. numazuensis* (Nishijima et al., 2013), and *E. montiporae* from (Yang et al., 2010) from the sea slug *Elysia ornate,* a sponge, and the coral *Montipora aequituberculata*, respectively), included genes for several forms of nitrate reductases, which would allow the conversion of nitrate to nitrite, and further the conversion of nitrite to ammonia. Neave et al. (2017a) further reported that the examined *Endozoicomonas* genomes all contained pathways for the assimilation of ammonia through the synthesis of glutamine and glutamate. However, this exciting hypothesis on the important putative function of *Endozoicomonas* awaits further experimental confirmation.

## Data Availability Statement

Sequence data determined in this study are available under NCBI BioProject ID PRJNA549164 (https://www.ncbi.nlm.nih.gov/bioproject/PRJNA549164). Abundant *Tridacna maxima* bacterial microbiome OTU reference sequences are available under GenBank Accession numbers MN108325 to MN10848 (https://www.ncbi.nlm.nih.gov/nuccore/?term=MN108325%3AMN10848%5BAccession%5D).

## Author Contributions

SR, AC, GP, CD, and CV designed and conceived the study. SR and GP generated data. SR, AC, CD, and CV analyzed the data. AC, GP, CD, and CV contributed reagents/tools/materials. SR, AC, CV wrote the manuscript. All authors contributed to improving the manuscript, read and approved the final manuscript.

### Conflict of Interest

The authors declare that the research was conducted in the absence of any commercial or financial relationships that could be construed as a potential conflict of interest.
