# Supplementary material for: Tissue-Specific Microbiomes of the Red Sea Giant Clam Tridacna maxima Highlight Differential Abundance of Endozoicomonadaceae
Source: Front Microbiol. 2019 Nov 26;10:2661. doi: 10.3389/fmicb.2019.02661 (PMC6901920; doi:10.3389/fmicb.2019.02661)
Supplement: Supplementary file 1 [file Data_Sheet_1.docx]

Supplementary Material

# Supplementary Figures


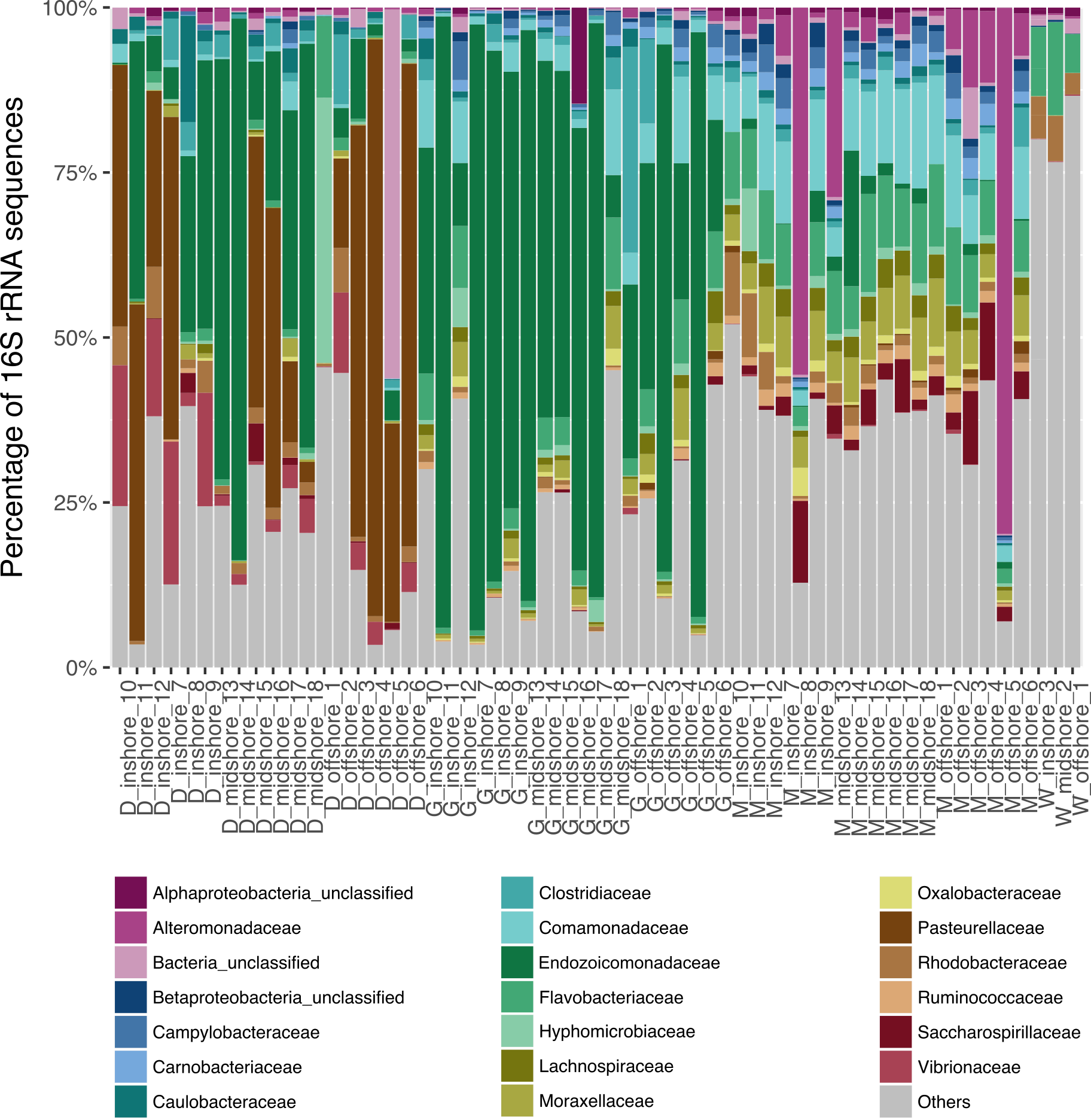


**Supplementary Figure 1.** Sample-based taxonomy bar plot of bacterial communities associated with *T. maxima* tissues. Bar plots show averaged relative abundances of the 20 most abundant families for the three tissue compartments (digestive system - D, gills - G, and mantle - M) over reef sites (inshore, midshore, offshore) and seawater (W) samples from the three reef sites.


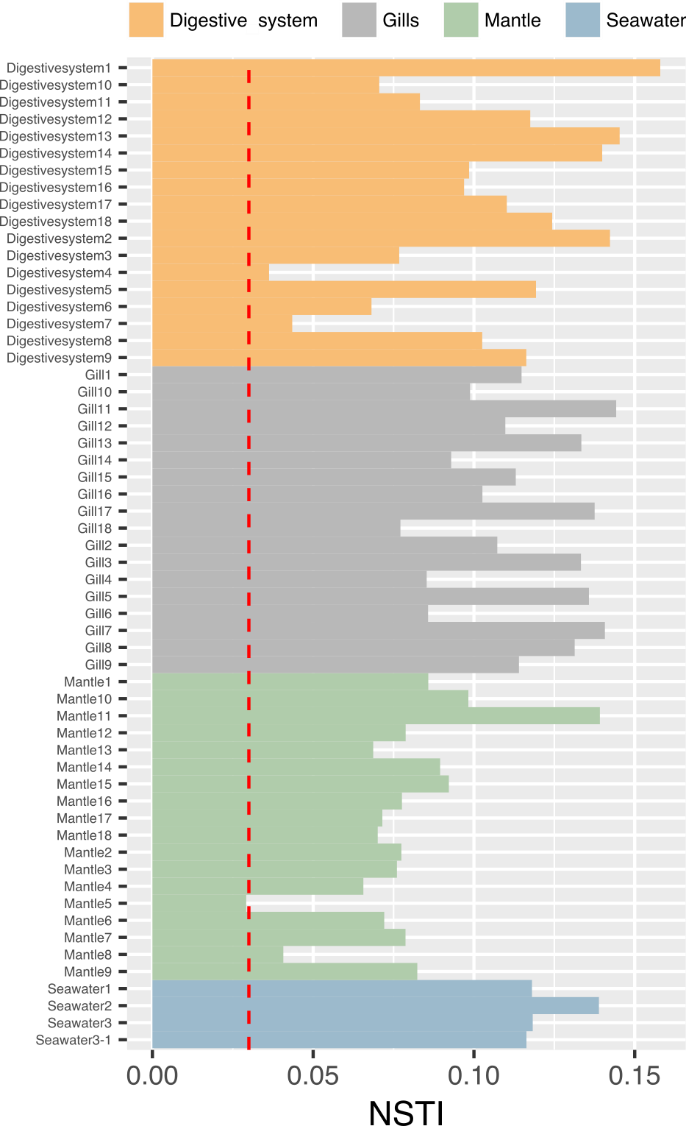


**Supplementary Figure 2.** PICRUSt-associated nearest Sequenced Taxon Indexes (NSTI).
Red dotted line represents predictive functional profiling cutoff at the species level (0.03 16S rRNA similarity). Functional inferences are based on similarities drawn between genus and family level.

**
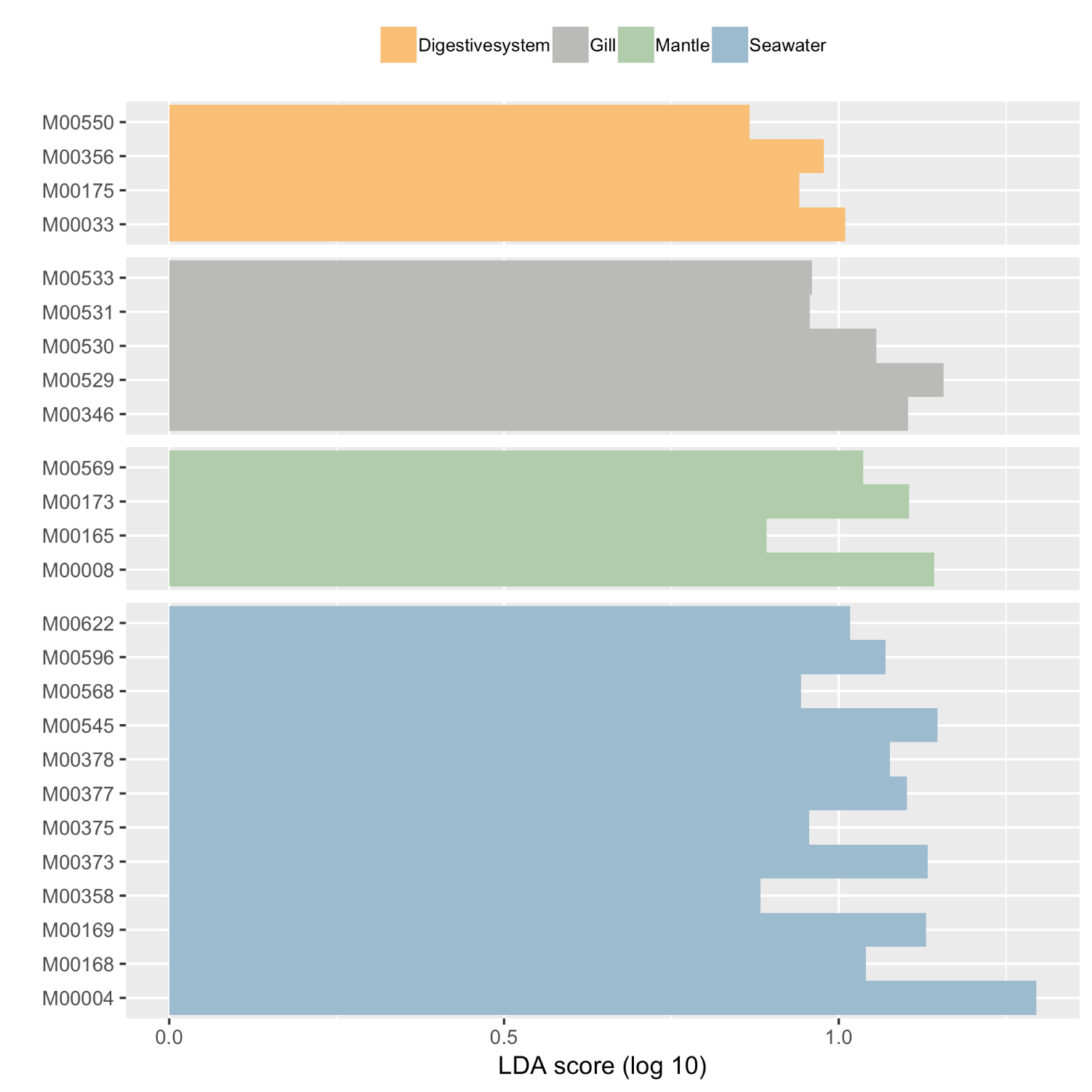
**

**Supplementary Figure 3.** The 25 modules from reference pathway "Microbial metabolism in diverse environments" (map01120) with effect sizes > 2.
